# 3D-printed origami electronics using percolative conductors†

**DOI:** 10.1039/c8ra04082f

**Published:** 2018-06-20

**Authors:** Yejin Jo, Du Won Jeong, Jeong-O Lee, Youngmin Choi, Sunho Jeong

**Affiliations:** Division of Advanced Materials, Korea Research Institute of Chemical Technology (KRICT) 19 Sinseongno, Yuseong-gu Daejeon 305-600 Korea youngmin@krict.re.kr sjeong@krict.re.kr; Department of Chemical Convergence Materials, Korea University of Science and Technology (UST) 217 Gajeongno, Yuseong-gu Daejeon 305-350 Korea

## Abstract

Recently, three-dimensional (3D) printing has garnered tremendous amounts of attention in various applications. In this study, we suggest a facile means of creating 3D-printed foldable electrodes on paper *via* the direct printing of composite pastes consisting of conductive fillers and a thermoplastic elastomer. The 3D-printability of the prepared composite pastes is investigated depending on the rheological properties. It is revealed that the composite paste with a high storage modulus would enable the formation of highly conductive features with a resistance of 0.4 Ω cm^−1^ on three-dimensional paper structures. The mechanical bending/folding stability levels of the printed electrodes are evaluated to judge the possibility of realizing 3D-printed origami electronics. The resistance is changed slightly with a normalized resistance value of 2.3, when the printed electrodes are folded with a folding angle of 150°. It is demonstrated that the 3D-printed composite electrodes are applicable to various origami electronics, including electrical circuits, strain sensors and electrochemical sensors.

## Abstract 2

Recently, three-dimensional (3D) printing has garnered tremendous amounts of attention in various applications.

## Graphical abstract

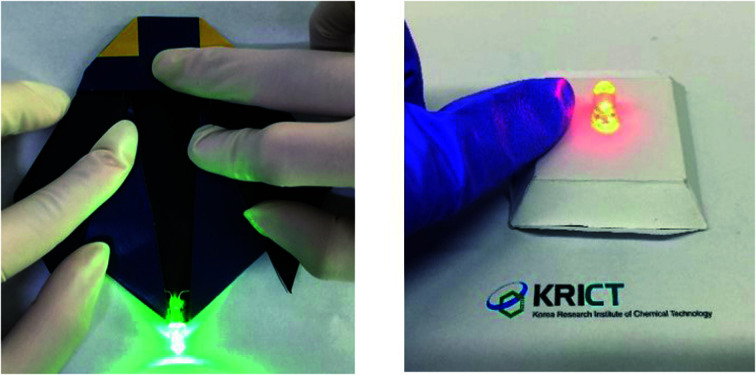

## Introduction

During the last decade, two-dimensional (2D) printed electronics devices have attracted much attention, as a variety of active/passive devices are capable of being fabricated without the use of conventional vacuum deposition and photolithographic patterning processes.^1–4^ As a next-generation technology, researchers have recently been attracted to three-dimensional (3D) printed electronics given the potential for various applications that cannot be realized with traditional 2D printing techniques.^5,6^ A resin-bath-free, directly-writable 3D printing technique possesses an advantage over its counterpart methods because it does not cause any damage to the underlying electrical components, unlike micro-stereolithography (SLA), dynamic-optical-projection stereolithography and selective laser sintering processes. To date, the directly writable 3D-printing process has shown extendable applicability in various areas, including strain sensors,^7^ lithium-ion batteries,^8^ light-emitting diodes,^9^ high-temperature heaters^10^ and biomedical applications.^11^ Origami paper electronics has not been suggested in the form of a 3D-printed structure.^12,13^ This can be accomplished if using 3D-printable, extremely foldable, highly conductive electrodes. Recently, we have reported a facile way of formulating 3D-printable, conductive composite paste comprising of silver flakes and multi-walled carbon nanotubes decorated with aqueous silver nanoparticles, with a usage of simple dispensing technique;^14^ but, an applicability toward 3D-printed origami electronics has not been suggested with a demonstration of foldable electrodes on paper substrates.

In this study, we demonstrate a simple means of fabricating 3D-printed origami devices through a direct-writable dispensing process of composite pastes consisting of conductive fillers and a thermoplastic elastomer. Highly conductive 3D-printable electrodes are formed on paper by regulating the rheological properties of the composite pastes. This is enabled by complete solvent evaporation toward the outward environment from the paper-printed structures, without capillary-force-driven migration into the pores present inside the paper. Apart from high conductivity, other prerequisites for origami electronics are achievable by the incorporation of a thermoplastic styrene-isoprene-styrene (SIS) tri-block copolymer as a matrix polymer in percolative composite electrodes. Given its unique chemical structure, the SIS tri-block copolymer endows the following functionalities to 3D-printed electrodes: (i) extreme flexibility due to its low elastic modulus, (ii) good adhesion due to the presence of a partially melted phase, and (iii) waterproofness as required for electrochemical sensor applications. It is demonstrated that the directly printed 3D electrodes are applicable to electrical circuits, strain sensors, and electrochemical sensors in the form of origami devices.

## Results and discussion

The composite pastes were formulated by mixing uniformly multi-component materials of Ag flakes as a primary conductive filler, Ag nanoparticle-decorated multi-walled carbon nanotubes (MWNTs) as a secondary conductive filler, the aforementioned SIS tri-block copolymer as a matrix polymer, and a solvent, as reported in our previous study.^14^ When the pastes are printed on dense polyethylene terephthalate (PET) plastic substrates, conductivity exceeding 23 000 S cm^−1^ is obtainable based on percolation conduction. Note that here the direct-writable dispensing process was carried out at room temperature and the printed structures were dried at room temperature as well merely to evaporate the solvents in the printed structures. Additional thermal processes were not applied to induce any cross-linking reaction of the matrix polymers and inter-particular sintering reactions between neighboring fillers. This implies that the composite pastes suggested in this study are printable on all types of paper substrates without consideration of the thermal budget.

**Fig. 1:**
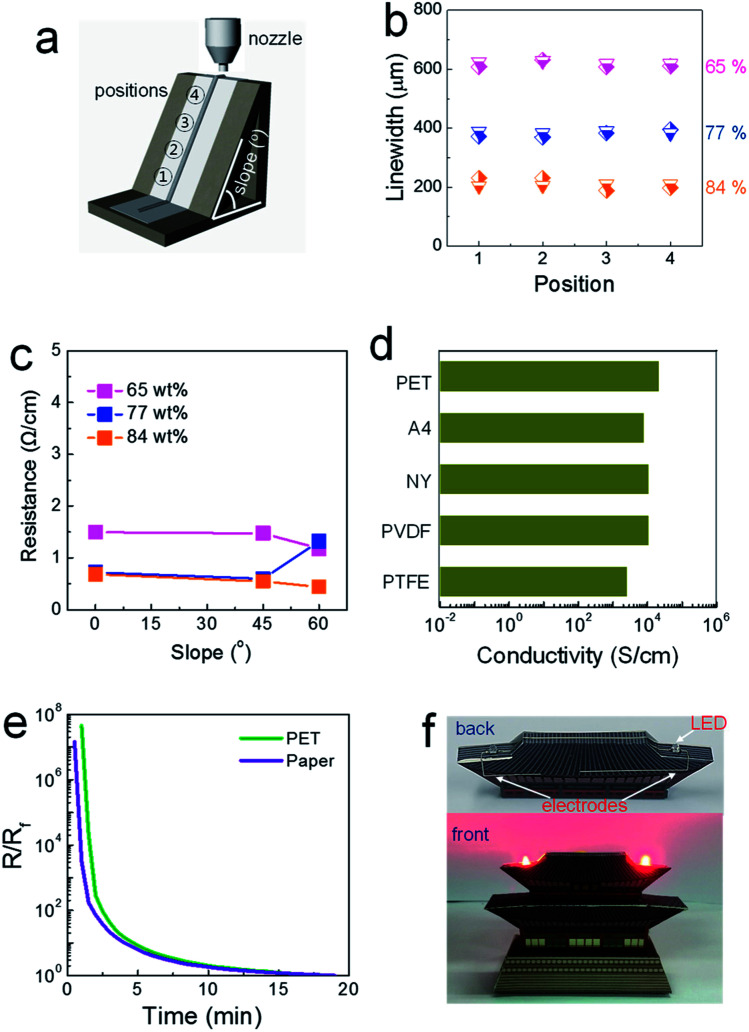
(a) Schematic of showing the printing process on papers attached to SLA-printed 3D structures. (b) Variation in the linewidth, and (c) variation in the resistance of electrodes printed on papers attached to SLA-printed 3D structures. In (b), the diamond and inverted triangle indicate the linewidths for electrodes printed on 45- and 60° sloped structures. (d) Conductivity values of electrodes printed on PET and various paper substrates. (e) Time-dependent change in the resistance during the drying process at room temperature for electrodes printed on PET and regular A4 substrates. Here, *R*_f_ denotes the resistance value for the finally dried electrodes. (f) Photographs of paper structures including 3D-printed electrodes.

The rheological properties of the composite pastes were regulated by adjusting the solid loading from 65 to 84 wt%. As shown in Table S1,† the storage moduli and viscosities were tailored with the values of 2900 and 578, 32 000 and 4920, and 68 500 Pa and 12 300 Pa s for the composite pastes with solid loadings of 65, 77 and 84 wt%, respectively. Composite pastes depending on the solid loading percentage are all denoted as “solid loading” wt% composite paste (for example, 84 wt% conductive paste) in this study. Initially, composite pastes with different rheological properties were printed on regular A4 paper attached to SLA-printed 3D structures, as shown in Fig. 1a. The inclined structure was designed to have a slope of either 45 or 60°. It was observed that the linewidths of the printed structures are invariant at all positions, even with the 65 wt% composite paste (Fig. 1b). The viscosity of the 65 wt% composite paste is higher by approximately by a factor of ∼10 than that of a conventional 2D-printable paste. Narrower patterns were generated by printing with more concentrated pastes with higher storage moduli, as a liquid-like flow is suppressed more in as-printed structures.

In contrast, the electrical properties varied significantly depending on the solid loading level, as shown in Fig. 1c. In electrodes printed using the 65 wt% composite paste, greater resistance was measured for patterns printed on flat and inclined structures compared to those printed with the 84 wt% composite paste on such structures. The paper used in this study is composed of macropores with an average diameter of 21.9 μm (Fig. S1†). When the paste with a low storage modulus is placed on top of a porous paper substrate, capillary-force-driven fluid migration occurs inside the macropores. In paper-based printed electronics, the suppression of fluid migration is a critical issue that must be dealt with to facilitate high-performance devices, as it critically degrades the electrical properties of printed layers.^15^ An additional passivating process is commonly employed to block the macropores existing in paper substrates.^15^ For the 84 wt% composite paste, a limited amount of solvent is incorporated with solid-like rheological characteristics (indicated by the extremely high storage modulus and viscosity), which restricts this type of migration behavior. A well-formed thick electrode structure, with an aspect ratio of 0.71, is shown in Fig. S2.† When the solid loading increased to an intermediate level of 77 wt%, low resistance values were measured for electrodes printed on a flat structure and an inclined structure with a slope of 45°; however, the resistance increased on the inclined structure with a slope of 60°, similar to when the 65 wt% composite paste was used, owing to the insufficient rheological properties represented by a slightly low storage modulus. Note that resistance of 0.4 Ω cm^−1^, obtainable in electrodes printed from the 84 wt% composite paste, is low enough to meet most specifications of practical electrical circuits.

Solvent evaporation without the involvement of capillary-force-driven migration could be confirmed by the conductivity values for structures printed on different types of paper and PET substrates. Solvent migration inside underlying pores is affected critically by the pore size and surface energy of the paper, as the capillary force is inversely proportional to the pore diameter and proportional to the surface energy of the substrate.^16^ However, as shown in Fig. 1d, the conductivity levels of electrodes printed with the 84 wt% composite paste did not change significantly when different types of paper substrates were used. Cellulose-based regular A4 paper is hydrophilic, and both polyvinylidene fluoride (PVDF) and polytetrafluoroethylene (PTFE) paper types are hydrophobic. The plastic PET substrate used here is free of pores and the paper substrates are composed of differently sized pores with diameters ranging from 1.9 to 110.5 μm (Fig. S1†). This characteristic drying behavior is also clarified through variations in the resistance as a function of the drying time. If both solvent evaporation and fluid migration occur simultaneously on porous paper substrates, the conductive electrodes should be generated nearly instantly on paper compared to the speed on a dense PET substrate. As shown in Fig. 1e, the trends of the reduced resistance are almost identical in the electrodes printed on porous A4 paper and on dense PET substrates. These results clarify how highly conductive electrodes can evolve through outward solvent evaporation from structures in a suspended form positioned on highly porous substrates. The capability of forming three-dimensionally printed electrode features is demonstrated in Fig. 1f. The highly conductive electrodes printed on structured paper substrates were barely reported to date, due to an absence of chemical strategy for preserving the conductive pathways inside structures printed on porous paper substrates. The resistance as low as 0.4 Ω cm^−1^, obtained in this study with 84 wt% conductive paste, was accomplished with following issues: (i) a usage of highly conductive paste that can allows for the electrical conductors with a conductivity over 23 000 S cm^−1^, (ii) an efficient suppression of capillary force-driven migration toward the pores residing in paper substrates, and (iii) a capability of forming the conductors with a high aspect ratio. In particular, the conductivity value measured in our 3D-printed electrodes is quite similar to those obtainable in percolative electrodes formed from commercially-available, screen-printable conductive pastes.^17^

**Fig. 2:**
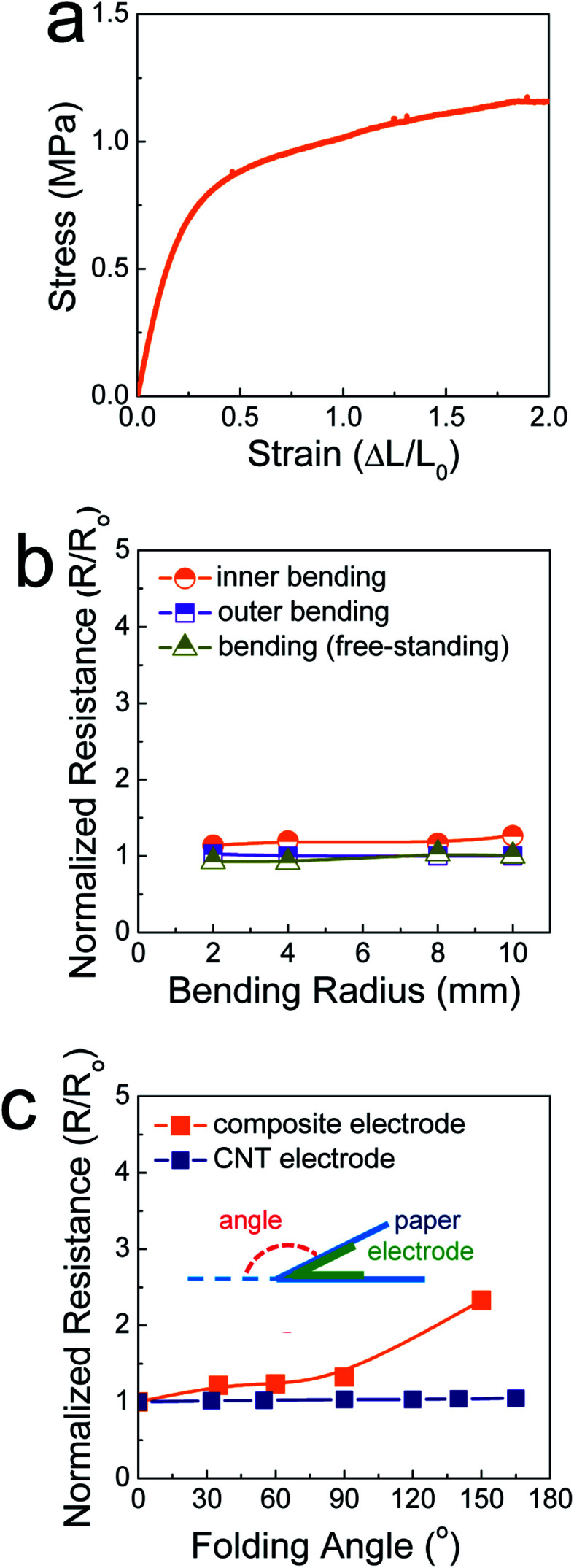
(a) Stress–strain curve of the SIS tri-block copolymer. (b) Variation in the normalized resistance as a function of the bending radius for electrodes printed on regular A4 paper using the 84 wt% composite paste, and (c) variation in the normalized resistance as a function of the folding angle for electrodes printed on regular A4 paper using the 84 wt% composite paste and MWNT paste.

Fig. 2a shows the stress–strain curve of the SIS tri-block copolymers. The SIS films could be stretched over a strain level of 2 without mechanical rupturing. The elastic modulus was measured and found to be 3.2 MPa. This value is comparable to that of poly(dimethylsiloxane) (PDMS), commonly used as a stretchable elastomer. When the ratio of the pre-polymer to the curing agent is 10 for PDMS, the elastic modulus is ∼0.7 MPa.^18^ In this study, PDMS was not feasible as a matrix polymer of composite pastes, as a polymerization reaction occurs even at room temperature. The rheological properties of PDMS-based composite paste change at a slow rate, resulting in uncontrollability of the 3D printing process. When the electrodes printed on 106 μm-thick A4 paper were stretched and compressed by outer and inner bending tests, they did not show significant changes in their resistance, similar to the case of free-standing, printed electrodes (Fig. 2b). This implies that the SIS elastomer-based, 3D-printed composite electrodes accommodate both tensile and compressive strain applied under a bending radius of 2 mm. For the printed electrodes folded with a folding angle up to 150°, the resistance increased slightly, with the normalized resistance being 2.3 (Fig. 2c and S3†). It is believed that the stable mechanical properties are due to the role of the secondary filler, Ag NP-decorated MWNTs, as well as the aforementioned role of the elastic SIS tri-block copolymer. When the composite electrodes are deformed mechanically, the conductive networks consisting of metallic flakes should be demolished to some extent. However, long, flexible one-dimensional conductive materials can preserve conductive networks^19–21^ as long as they interact with primary metallic fillers through chemical bonds. In our composite electrodes, the Ag flakes undergo coordination bonding with the carboxyl groups present on the surface of the Ag NPs in the Ag NP-decorated MWNTs.^14^ As presented in Fig. 2c and S4,† metallic flake-free electrodes composed of pristine MWNTs and the SIS copolymer exhibits extremely stable electrical properties during identical folding tests. However, the conductivity is limited to 9.3 S cm^−1^, implying that these carbon-nanotube-based electrodes are scarcely applicable to high-performance electrical circuities.

**Fig. 3:**
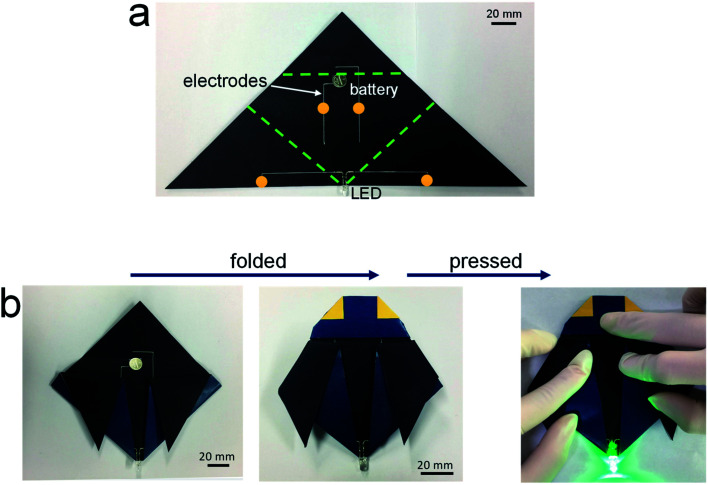
(a) Photograph of electrical circuits printed on black-colored paper, and (b) photographs showing the origami procedure used with the printed circuits. The fabricated sample was folded two times sequentially along the green dashed lines, by which the open-circuit electrodes came into contact at the areas denoted by the yellow circles.

**Fig. 4:**
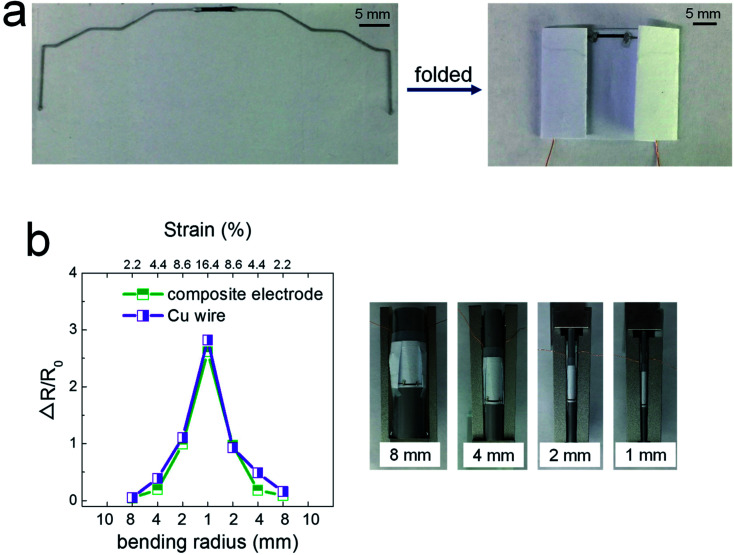
(a) Photographs of strain-sensor devices employing foldable electrodes printed on regular A4 paper, and (b) resistance signal as a function of bending radius in a strain-sensor device with either a folded printed electrode or un-folded Cu wire.

Fig. 3 shows the potential applicability to paper-based origami electronics. The 84 wt% composite paste was printed to form electrodes on black-colored paper, with both a light-emitting diode (LED) and a battery attached (Fig. 3a). As shown in Fig. 3b, the fabricated sample was folded two times sequentially along the green dashed lines, allowing the open-circuit electrodes to come into contact in the components circled in yellow. When external pressure was applied to form firm electrical contact, the circuit is activated and the LEDs turn on. For a more practical demonstration, we fabricated strain-sensor devices consisting of a strain-sensitive piezoresistive layer and electrodes (Fig. 4). Both layers were formed by a printing process. The piezoresistive layer was printed using pastes prepared from a mixture of hybrid carbons and SIS tri-block copolymer, as described in our previous report.^22^ The electrode layers were printed using the 84 wt% composite paste. Both end parts of electrodes were folded completely, as shown in Fig. 4a, and the change of the normalized resistance was then measured while the devices were bent at different outer bending radii ranging from 1 to 8 mm. It was clearly observed that the resistance signal varies depending on the tensile strain level applied during the bending tests (Fig. 4b). As a control sample, strain-sensor devices composed of a printed sensor layer and unfolded Cu wires were tested under identical deformation conditions. There were no significant differences in the resistance signals of both devices. If the folded electrodes underwent a significant resistance change, the sensing signal of the printed devices should differ from that of devices based on unfolded Cu wire. These results suggest that electrodes printed from the 84 wt% composite paste can be applied to structurally deformable origami device architectures. Note that the printed electrodes do not undergo any delamination from the paper substrates during repeated folding and bending tests. Good adhesion is one of prerequisites of printing-based origami electronics. The well-controlled adhesion properties demonstrated here are attributable to the characteristic chemical structure of the SIS tri-block copolymer. The isoprene segment with a glass transition temperature below −60 °C can be partially melted even at room temperature, which contributes critically to the formation of the suitably adhesive printed structures.^14^

**Fig. 5:**
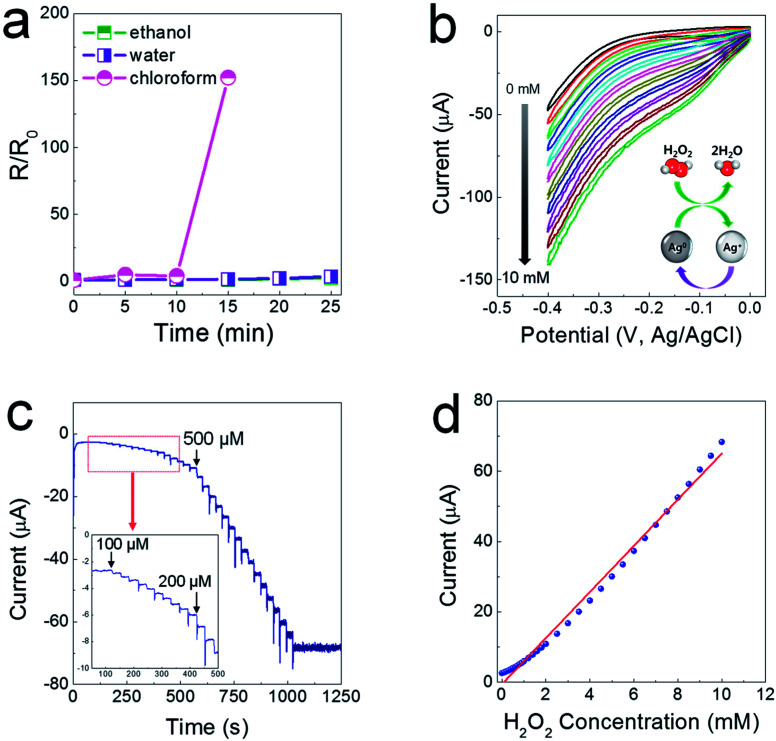
(a) Time-dependent variation in resistance for electrodes immersed in various solvents, in this case ethanol, water, and chloroform. (b) Cyclic voltammetry data at a scan rate of 20 mV s^−1^ for Nafion-wrapped composite electrodes in a 10 mM PBS (pH 7.4) solution when increasing the concentration of H_2_O_2_ from 1 to 10 mM. (c) Amperometric response of a Nafion-wrapped composite electrode at a potential of −0.2 V with successive additions of 100, 200, and 500 μM H_2_O_2_ in a stirred 10 mM PBS solution. (d) Calibration plot corresponding to the amperometric response of H_2_O_2_.

As another characteristic of electrodes printed from composite pastes, they are sustainable in a polar solvent medium, as the SIS tri-block copolymer is soluble only in non-coordinating solvents. As shown in Fig. 5a, when the printed electrodes were immersed completely in ethanol and water baths, the resistance levels of the printed electrodes did not change for a prolonged period of time. For the electrodes immersed in a chloroform bath, the resistance soared abruptly after 15 min, showing insulating properties after 20 min. This water-proof property could allow for the possibility of fabricating electrochemical sensor devices. It has been reported that Ag nanoparticles (NPs) exhibit excellent electrocatalytic activity when used to reduce hydrogen peroxide (H_2_O_2_)^23–25^ generated from biochemical reactions catalyzed by a glucose oxidase enzyme. To verify the electrocatalytic activity, cyclic voltammetry (CV) was utilized in a low negative potential range of −0.4–0 V to prevent the flow of impeding current by electrochemical reactions between the Ag phase and the chlorine ions contained in a phosphate-buffered saline (PBS) solution (0.01 M phosphate, 0.0027 M potassium chloride and 0.137 M sodium chloride, pH 7.4). The *CV* curves show that the cathode current increases linearly depending on the concentration of H_2_O_2_ from 1 to 10 mM, indicative of excellent reproducibility and stability of the electrocatalytic reaction (Fig. 5b). As presented in Fig. 5c, the chronoamperometric current increased stepwise with a successive addition of H_2_O_2_ at a potential of −0.2 V, and a steady-state current was obtained within 8–9 s. The amperometric response showed a linear relationship in concentrated H_2_O_2_ from 100 μM to 10 mM, with sensitivity of 6.55 μA mM^−1^ and a correlation coefficient of 0.99 (Fig. 5d).

**Fig. 6:**
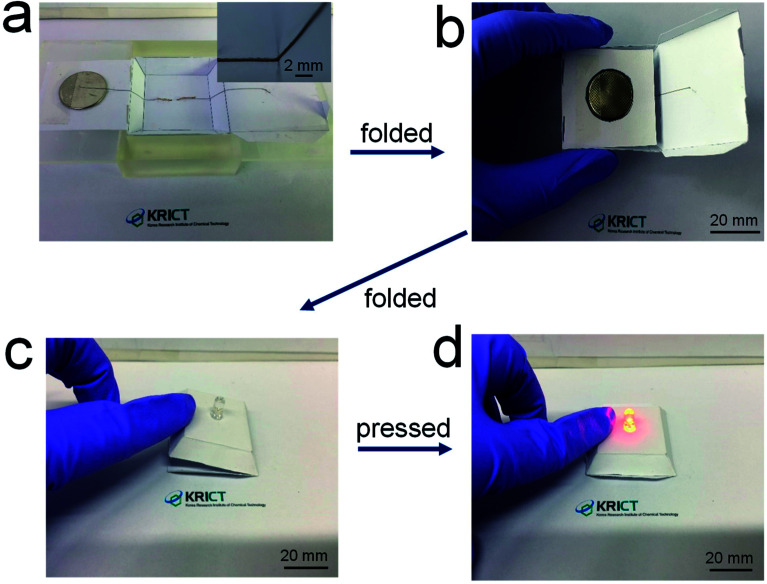
(a) Photograph of electrical circuits including electrodes 3D-printed along the surface of a paper structure, and (b)–(d) photographs of origami-processed electrical circuits.

Fig. 6 shows demonstration of the 3D-printed, origami electrical circuits. The paper substrates were placed on a SLA-printed template. A battery was loaded onto the surface of the paper substrate and the LED was positioned beneath the paper substrate. The 84 wt% composite paste was printed in conformal contact with the LED terminals and along a steep slope of the pre-formed paper structure, not leaving any spaces between the printed electrode and the substrate (Fig. 6a). A side wing with the battery was then folded (Fig. 6b), and another side wing with the electrode was subsequently folded on top of a side of the battery, thus creating a fully folded origami structure employing internal electrical circuits (Fig. 6c). When gentle pressure was applied, the electrode located on the bottom side of the complete origami structure came into contact with the battery, which activated the electrical circuit and turned on the LED (Fig. 6d). The repeated operation of this is shown in Movie S1.† These results suggest that the composite paste allows for the formation of highly conductive, printed foldable electrodes, thus providing a new possibility of fabricating 3D-structured origami devices in a versatile form in electronics and electrochemical applications.

## Conclusion

In summary, here we formulated a composite paste consisting of multi-component conductive fillers, an elastomeric SIS tri-block copolymer, and a small amount of solvent. It was revealed that the 3D-printability and conductivity are well regulated depending on the rheological properties of the composite pastes. In addition, it was found that good mechanical stability is obtainable by a virtue of the combined roles of the Ag NP-decorated carbon nanotubes and the elastomeric matrix. Using electrodes printed from highly viscous composite pastes, various origami devices were demonstrated successfully, including electrical circuits, strain sensors and electrochemical sensors.

## Experimental

### Preparation of the composite pastes

The composite pastes were formulated by the methods described in our previous report.^14^ For the synthesis of amine-functionalized, multi-walled carbon nanotubes (NH_2_MWNTs), a mixture composed of 1.4 g of MWNT (97%, length: about 10 μm, Applied Carbon Nano Co. Ltd.), 0.35 g of perylene-3,4,9,10-tetracarboxylic dianhydride (PTDA, 97%, Aldrich), 350 mL of methylene chloride (99.5%, Samchun), 70 mL of triethylamine (99%, Samchun), and 14 mL of ethylenediamine (≥99%, Aldrich) is sonicated for 1 h and stirred vigorously for 24 h. The mixture is centrifuged and the precipitate is washed with methanol, methylene chloride and methanol (in that order) by centrifugation, followed by drying in a vacuum overnight. The poly(acrylic acid)-capped silver nanoparticles are synthesized by chemical reduction of Ag ions in DI water. To prevent agglomeration, polyvinylpyrrolidone (PVP, average MW ∼ 10 000) and poly(acrylic acid) (PAA, sodium salt, average *M*_w_ ∼ 15 000, 35 wt% in H_2_O) are incorporated as surface-capping molecules and sodium borohydride is used as a reducing agent. In a typical procedure, 4.7 g of Ag nitrate (99.9%, Kojima Chemicals), 3.8 g of poly(acrylic acid), and 6.0 g of PVP were added to a three-necked, round-bottom flask containing 100 mL of DI water at a pH of 11. The resultant solution is heated to 60 °C and stirred with a magnetic stirrer under a refluxing condition. When the temperature reaches 60 °C, 9.7 g of an aqueous sodium borohydride (98.5%, Kojima Chemicals) solution (pH = 11) is injected. After reacting for 60 min at 60 °C, the synthesized Ag nanoparticles are selectively separated by centrifugation, followed by washing by DI water. To decorate the NH_2_MWNTs with the PAA-capped Ag nanoparticles, 50 g of an aqueous NH_2_MWNT solution with a concentration of 3 mg mL^−1^ is mixed with 6.75 g of an aqueous poly(acrylic acid) capped-Ag NP solution at a concentration of 20 wt%. After a subsequent sonication/homogenization process, the mixture is centrifuged to collect the PAA-Ag/NH_2_MWNT hybrid material. The precipitates are then dispersed in DI water, with the pH then adjusted to 4. After the centrifugation process, the precipitates are re-dispersed in ethyl alcohol, followed by centrifugation. The resultant precipitates are then mixed with a proper amount of Ag flakes (SF120, Ames Advanced Materials Corporation) and 50 g of toluene. After centrifugation, the precipitates are mixed manually with a proper amount of polystyrene–polyisoprene–polystyrene (SIS, styrene 22%, 12 poise @ 25 wt% in toluene, Aldrich) and 1,3-dichlorobenzene (≥99.0%, Aldrich) in an agate mortar for 3 min. The weight fraction of conductive fillers to SIS is kept constant at 0.94 and the total solid loading is varied from 65–84 wt%.

### Printing of the composite pastes

The composite pastes were printed using a programmable dispenser (Image Master 350PC Smart, Musashi) and a nozzle with an inner diameter of 350 μm. The printing speed and back air pressure were 1–10 mm s^−1^ and 100–200 kPa, respectively. The printed patterns were dried at room temperature. The stereo-lithographically (SLA) printed polymer structures were fabricated using a 3D printer (Form 2, Formlabs). To create the paper-based 3D structures, regular A4 paper (Hankuk Paper Mfg, square mass: 75 g m^−2^) was attached onto the surfaces of the SLA-printed polymer structures. As a plastic substrate, a 125 μm-thick polyethylene terephthalate (PET, Tetron KEL 86w, DuPont) substrate was used. As other paper substrates, mixed cellulose esters (MCE, MCE02047A, Hyundai Micro), nylon (NY, NY020047A, Hyundai Micro), poly(vinylidene fluoride) (PVDF, PVDF2047A, Hyundai Micro) and polytetrafluoroethylene (PTFE, PTFE020047S, Hyundai Micro) membrane filter papers were used as received without a cleansing process.

### Fabrication of strain-sensor devices

The carbon paste for the strain-sensor devices was formulated by the method reported in our recent study.^22^ Briefly, NH_2_MWNTs were assembled on the surface of graphene oxides through electrostatic interaction, and the synthesized carbon hybrids were mixed with styrene-isoprene-styrene (SIS) tri-block copolymer and dichlorobenzene. The sensing layer was printed on regular A4 paper substrates and dried at 80 °C.

### Characterization

The morphologies of the printed patterns were observed with scanning electron microscopy (SEM, JSM-6700, JEOL). The electrical resistance levels were evaluated with an interactive digital source meter (2450, Keithley) by 4-probe measurement mode. The printed lines were connected with Cu wires (The Nilaco Corporation, diameter: 0.20 mm) by Ag epoxy (Chemtronics, CW2400) pads. The rheology properties of composite pastes were monitored using a modular compact rheometer system (MCR 101, Anton Paar). Cyclic voltammetry and amperometric responses were measured using an electro-chemical analyzer (SP-300 potentiostat, BioLogic) with a three-electrode electrochemical cell consisting of a Nafion-wrapped composite electrode (5 wt% in a mixture of lower aliphatic alcohols and water, Aldrich) as a working electrode, a platinum-coated titanium mesh as an auxiliary electrode, and an Ag/AgCl (3 M KCl) as a reference electrode.

## Conflicts of interest

There are no conflicts to declare.

## Supplementary Material

RA-008-C8RA04082F-s001

RA-008-C8RA04082F-s002
