# Supplementary material for: 3D-printed origami electronics using percolative conductors
Source: RSC Adv. 2018 Jun 20;8(40):22755–62. doi: 10.1039/c8ra04082f (PMC9081645; doi:10.1039/c8ra04082f)
Supplement: RA-008-C8RA04082F-s001 [file RA-008-C8RA04082F-s001.pdf]

Electronic Supplementary Material (ESI) for RSC Advances.
This journal is © The Royal Society of Chemistry 2018

Supplementary Information

3D-Printed Origami Electronics Using Percolative Conductors

Yejin Jo,[a,b] Du Won Jeong,[a] Jeong-O Lee,[a] Youngmin Choi,[a,b,*] Sunho Jeong[a,b,*]

[a]Division of Advanced Materials, Korea Research Institute of Chemical Technology (KRICT), 19 Sinseongno, Yuseong-gu, Daejeon 305-600, Korea

[b]Department of Chemical Convergence Materials, Korea University of Science and Technology (UST), 217 Gajeongno, Yuseong-gu, Daejeon 305-350, Korea

E-mail: youngmin@krict.re.kr (Y. Choi); sjeong@krict.re.kr (S. Jeong)

KEYWORDS: 3D, print, circuit, origami, percolative

Table S1. Values of the storage modulus and viscosity for the 65, 77 and 84 wt% composite pastes

	65 wt%	77 wt%	84 wt%
Storage Modulus (Pa)	2,900	32,000	68,500
Viscosity (Pa·s)	578	4,920	12,300

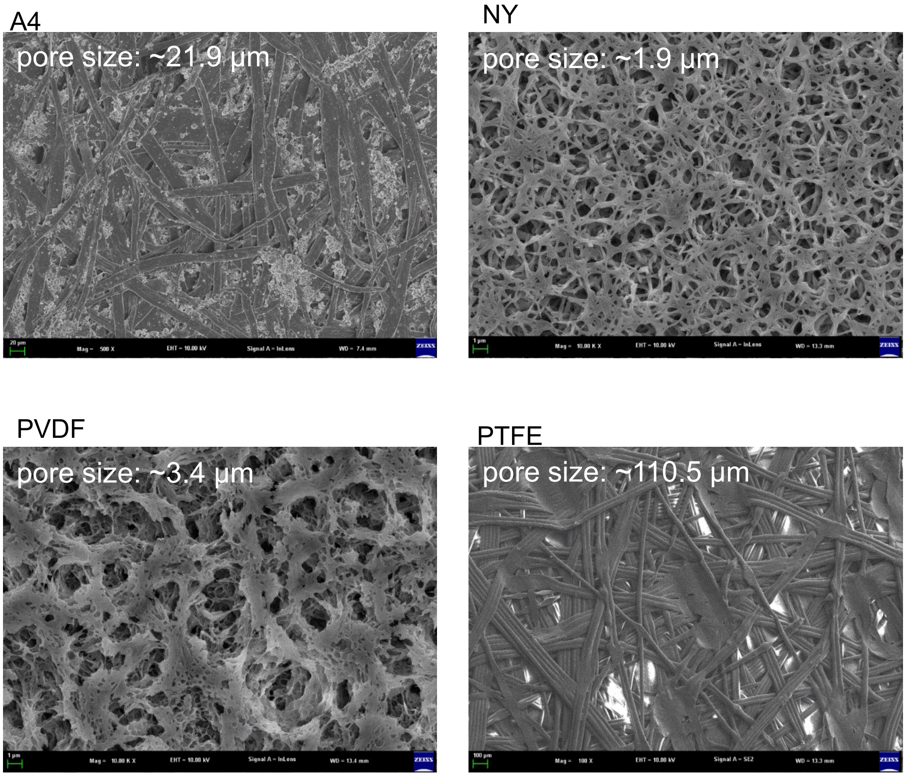

Figure S1. SEM images of the paper types used in this study.

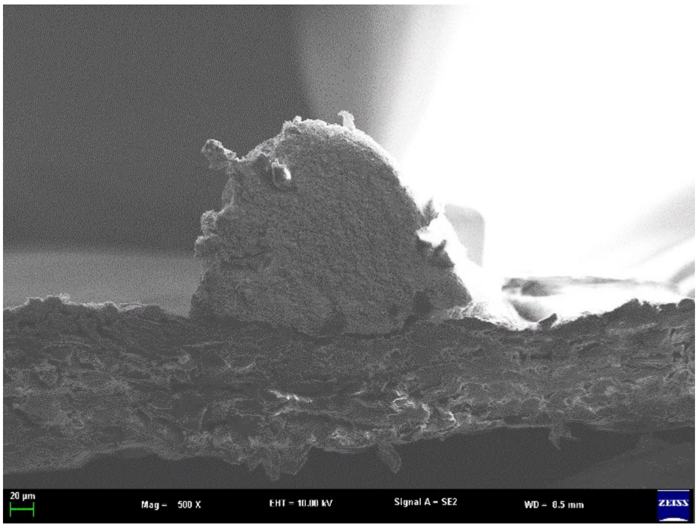

Figure S2. Cross-sectional SEM image of an electrode printed on regular A4 paper using the 84 wt% composite paste.

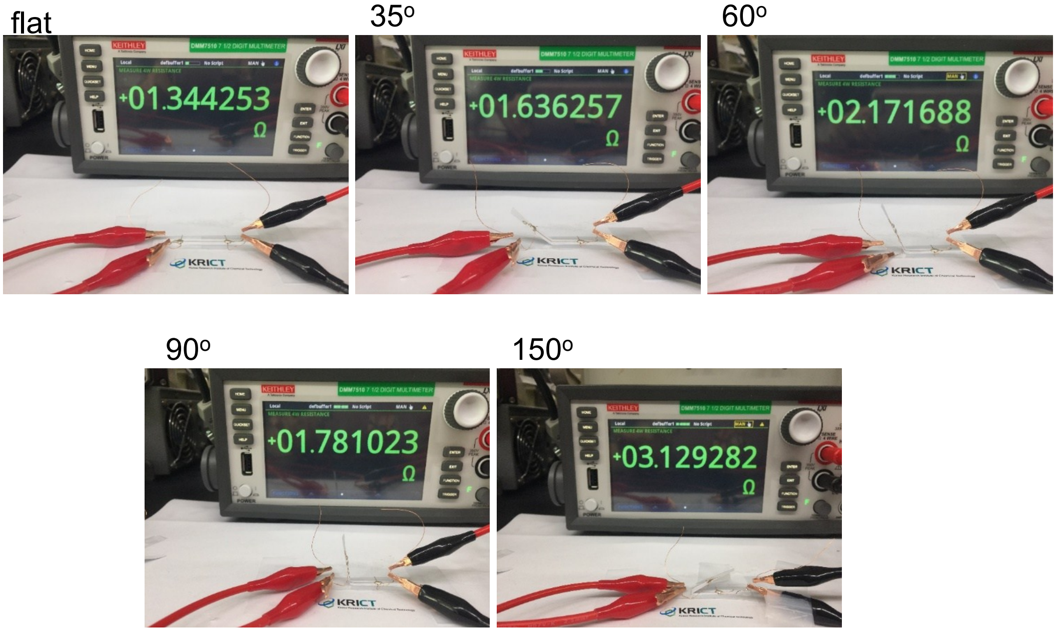

Figure S3. Photographs showing the measured resistance of electrodes printed on regular A4 paper using the 84 wt% composite paste.

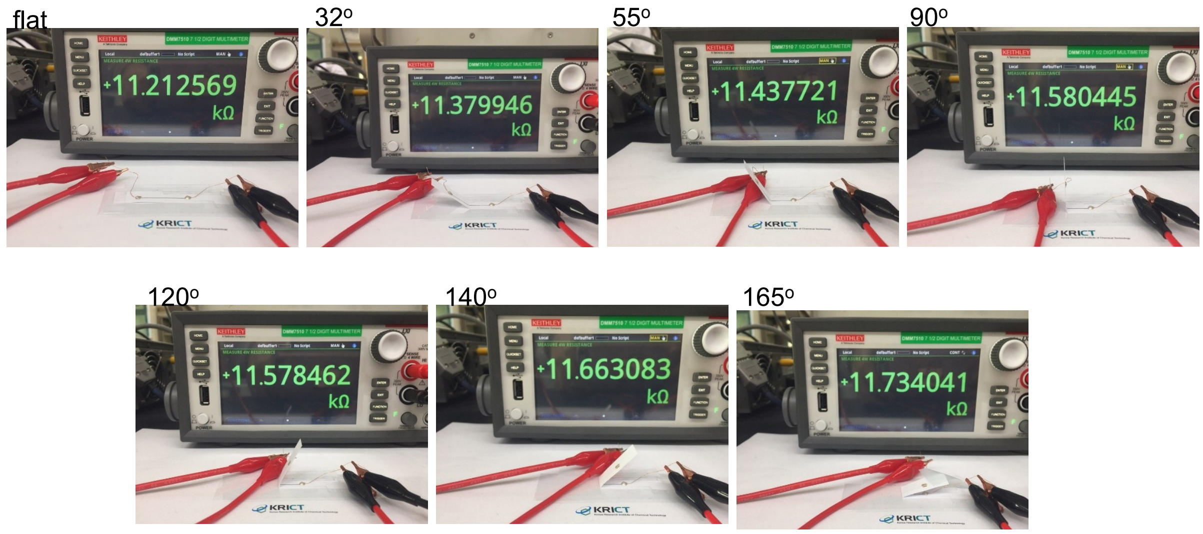

Figure S4. Photographs showing the measured resistance of electrodes printed on regular A4 paper using the MWNT paste.

Movie S1. Motion picture showing the operation of origami-processed electrical circuit.
